# Supplementary material for: Identification and functional analysis of non-coding regulatory small RNA FenSr3 in Bacillus amyloliquefaciens LPB-18
Source: PeerJ. 2023 May 15;11:e15236. doi: 10.7717/peerj.15236 (PMC10194069; doi:10.7717/peerj.15236)
Supplement: Supplemental Information 4 [file peerj-11-15236-s004.zip › KO/CK-vs-T1_map/map05010.html]

KEGG PATHWAY: Alzheimer disease - Reference pathway


|  |  |
| --- | --- |
| **Alzheimer disease - Reference pathway** |  |

[
Pathway menu
| Organism menu
| Pathway entry
| Show description
| User data mapping
]

Alzheimer disease (AD) is a chronic disorder that slowly destroys neurons and causes serious cognitive disability. AD is associated with senile plaques and neurofibrillary tangles (NFTs). Amyloid-beta (Abeta), a major component of senile plaques, has various pathological effects on cell and organelle function. The extracellular Abeta oligomers may activate caspases through activation of cell surface death receptors. Alternatively, intracellular Abeta may contribute to pathology by facilitating tau hyper-phosphorylation, disrupting mitochondria function, and triggering calcium dysfunction. To date genetic studies have revealed four genes that may be linked to autosomal dominant or familial early onset AD (FAD). These four genes include: amyloid precursor protein (APP), presenilin 1 (PS1), presenilin 2 (PS2) and apolipoprotein E (ApoE). All mutations associated with APP and PS proteins can lead to an increase in the production of Abeta peptides, specfically the more amyloidogenic form, Abeta42. FAD-linked PS1 mutation downregulates the unfolded protein response and leads to vulnerability to ER stress.

|  |  |  |
| --- | --- | --- |
| Reference pathway | 184% 150% 122% 100% 82% 67% 55% | 图片下载 |
